# The comparison of adipose-derived stromal cells (ADSCs) delivery method in a murine model of hindlimb ischemia

**DOI:** 10.1186/s13287-024-03634-2

**Published:** 2024-02-02

**Authors:** Ewelina Pilny, Justyna Czapla, Alina Drzyzga, Ryszard Smolarczyk, Sybilla Matuszczak, Magdalena Jarosz-Biej, Łukasz Krakowczyk, Tomasz Cichoń

**Affiliations:** 1 Center for Translational Research and Molecular Biology of Cancer, Maria Skłodowska-Curie National Research Institute of Oncology, Gliwice Branch, Wybrzeże Armii Krajowej Street 15, 44-102 Gliwice, Poland; 2 Department of Oncologic and Reconstructive Surgery, Maria Skłodowska-Curie National Research Institute of Oncology, Gliwice Branch, Wybrzeże Armii Krajowej Street 15, 44-102 Gliwice, Poland

**Keywords:** ADSCs, Mesenchymal stromal cells, Hindlimb ischemia, Muscle regeneration, Angiogenesis, Arteriogenesis, Macrophages

## Abstract

**Background:**

Adipose-derived stromal cells (ADSCs) demonstrate ability to promote tissue healing and down-regulate excessive inflammation. ADSCs have been used to treat critical limb ischemia in preclinical and clinical trials, but still, there is little known about their optimal delivery strategy. To date, no direct analysis of different methods of ADSCs delivery has been performed in the hindlimb ischemia model. Therefore, in this study we focused on the therapeutic efficacy of different ADSCs delivery methods in a murine model of hindlimb ischemia.

**Methods:**

For the hADSCs isolation, we used the subcutaneous adipose tissue collected during the surgery. The murine hindlimb ischemia was used as a model. The unilateral femoral artery ligation was performed on 10–12-week-old male C57BL/6. ADSCs were delivered directly into ischemic muscle, into the contralateral muscle or intravenously. 7 and 14 days after the surgery, the gastrocnemius and quadriceps muscles were collected for the immunohistochemical analysis. The results were analyzed with relevant tests using the Statistica software.

**Results:**

Our research revealed that muscle regeneration, angiogenesis, arteriogenesis and macrophage infiltration in murine model of hindlimb ischemia differ depending on ADSCs delivery method. We have demonstrated that intramuscular method (directly into ischemic limb) of ADSCs delivery is more efficient in functional recovery after critical limb ischemia than intravenous or contralateral route.

**Conclusions:**

We have noticed that injection of ADSCs directly into ischemic limb is the optimal delivery strategy because it increases: (1) muscle fiber regeneration, (2) the number of capillaries and (3) the influx of macrophages F4/80^+^/CD206^+^.

**Supplementary Information:**

The online version contains supplementary material available at 10.1186/s13287-024-03634-2.

## Background

The critical limb ischemia (CLI) is an advanced stage of a peripheral arterial disease, characterized by chronic limb resting pain with ulceration or tissue necrosis. As a result of atherosclerosis progression, it comes to stenosis and occlusion of arteries, so that the arterial blood supply to tissues of the lower extremities is almost completely limited. CLI affects 3% of the world's population and is associated with high morbidity and mortality: Every fifth patient dies within 6 months of diagnosis, and every second patient does not survive 5 years [1, 2]. Depending on the progression of disease or the patient's health condition, percutaneous interventions or coronary aortic bypass surgery is performed [3]. However, most patients, due to comorbidities or occluded blood vessels, do not qualify for revascularization. A promising treatment strategy for patients with no available treatment options become a therapeutic angiogenesis, wherein the proangiogenic growth factors or cells are used. The main purpose of this therapy is to promote new blood vessel development and improve ischemic tissue perfusion [4, 5].

In recent years, cell therapy utilizing mesenchymal stromal cells (MSCs) has become the subject of intense research in the field of treatment of patients with CLI. Both adipose-derived mesenchymal stromal cells (ADSCs) and bone marrow-derived mesenchymal stromal cells (BM-MSCs) were used in preclinical and clinical studies to treat CLI [6–9]. In our previous study, we observed better proangiogenic properties of ADSCs compared to BM-MSCs in murine model of hindlimb ischemia [10]. The hindlimb ischemia model involves acute disruption of blood supply to the arteries and is generally used as a preclinical method assessing angiogenic and arteriogenic regulation in peripheral arterial disease [11]. Also others demonstrated a better efficacy in the treatment of mice with CLI with ADSCs compared to MSCs isolated from the bone marrow [12, 13]. However, there are still many inaccuracies concerning the place, time or the number of delivered cells in proposed therapies for CLI.

To date, no direct analysis of different methods of ADSCs delivery has been performed in the hindlimb ischemia model. For this reason, the relationship between transplantation method and treatment efficacy is unclear. In our study, we tested three different methods of ADSCs delivery in a murine model of CLI to evaluate their potential in the hindlimb ischemia treatment. ADSCs were delivered: intramuscularly—into the gastrocnemius muscle of hindlimb where femoral artery ligation was performed, contralaterally—into contralateral limb, where femoral artery ligation was absent, or intravenously—into tail vein. To our knowledge, this is the first study evaluating different methods of ADSCs delivery for the CLI treatment.

## Methods

### Ethical statements

The study with the use of human adipose tissue was performed in accordance with the Declaration of Helsinki, with the approval of the Committee on the Bioethics. Abdominal subcutaneous adipose tissue was collected during planned surgery in Maria Sklodowska-Curie National Research Institute of Oncology, Gliwice Branch (Poland). Patients ranged in age from 24 to 64 years. Tissue was collected from 4 women and 2 men.

The animal treatment and the experimental procedures of the study were approved by the Committee on the Ethics of Animal Experiments of the Local Ethics Commission. All experiments on animals were conducted in accordance with the 3R rule and at the same time with taking into consideration the right number of the animals in groups making it possible to run the statistical analysis. Sample size was determined by power analysis, power set to 0.8 at 0.05 significance level.

More detailed information are included in the ethics approval and consent to participate section.

### Cell culture

Human adipose-derived stromal cells (ADSCs) were isolated, cultured and characterized as described elsewhere [10, 14, 15]. Briefly, human adipose tissue was cut into pieces and digested with collagenase NB4 solution (0.39 U/ml, Serva Electrophoresis, Heidelberg, Germany) for 1 h at 37 °C. Red blood cells were lysed (0.15 M ammonium chloride solution; Merck, Darmstadt, Germany). Then, single cell suspension was placed on plastic culture dishes (6 × 10^6^ cells/10 cm plate) in DMEM medium with high glucose concentration (4.5 g/L) (Life Technologies, Carlsbad, CA, USA) supplemented with 20% FBS (Gibco BRL, Paisley, UK) and antibiotics (penicillin and streptomycin, Merck, Darmstadt, Germany). Cells were incubated at 37 °C with 5% of CO_2_. The experiments were conducted after the second passage of the cells. Before every ADSCs injection, their immunophenotype was assessed according to the International Society for Cellular Therapy guidelines [10, 14, 16].

### Mouse model of hindlimb ischemia

**Scheme 1:**
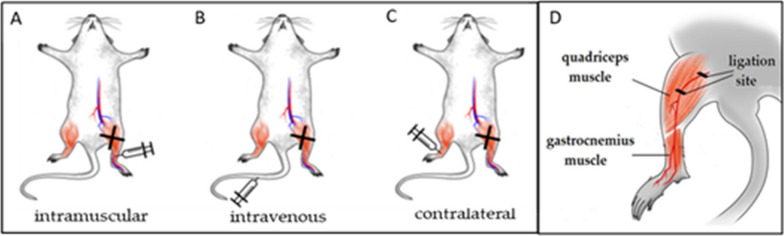
ADSCs delivery scheme in a murine model of hindlimb ischemia. **A** Intramuscular—ADSCs were injected into the gastrocnemius muscle of the limb where the artery ligation in quadriceps muscle was performed. **B** Intravenous—ADSCs were injected into tail vein. **C** Contralateral—ADSCs were injected into the gastrocnemius muscle of contralateral limb, where femoral artery ligation was absent (X-ligation site). **D** Schematic diagram of hindlimb ischemia model and the analyzed muscles

C57BL/6NCrl mice (eight- to twelve-week-old males) (*n* = 45) were originally purchased from Charles River Laboratories (Wilmington, MA, USA). All mice were housed in the Maria Sklodowska-Curie National Research Institute of Oncology, Gliwice Branch (Poland), in a pathogen-free facility in SPF standard. The unilateral femoral artery ligation was performed on mice as described previously [10, 14]. Briefly, animals were anesthetized with 2% isoflurane (MiniVent Model 845, Harvard Apparatus, USA), then the skin from the inner side of the left hindlimb was incised and the superficial femoral artery was exposed. The artery was ligated at two points using triple surgical knots. Skin incisions were closed. An hour after the ligation, 1 × 10^6^ of ADSCs suspended in 100 μL of PBS^−^ were delivered: (A) intramuscularly—into the gastrocnemius muscle of hindlimb where unilateral femoral artery ligation was performed, (B) intravenously—into tail vein and (C) contralaterally—into contralateral limb, where unilateral femoral artery ligation was absent (Scheme 1). The cells were administered with a single injection. Control mice were subjected only to femoral ligation procedure (ischemic). All treatment groups include *n* = 5 mice.

In experiments investigating muscle regeneration, angiogenesis and infiltration of macrophages, to evaluate the effect of ADSCs delivery method on the ischemic limbs, the gastrocnemius muscles were collected from the ligated limbs on the 7th and the 14th day post-injury (dpi). To assess arteriogenesis additionally, we investigated the quadriceps muscle (Scheme 1D). The control group (ischemic) consisted of mice in which only artery ligation was performed (represented physiological processes of ischemic tissue regeneration). Additionally, the non-ischemic muscles from healthy mice were collected (*n* = 5).

The detailed contributions of animal procedure presented in the study are included in Additional file 1. Further inquiries can be directed to the corresponding authors.

### Tissue preparation

On the 7th and the 14th days after surgery, mice were killed by cervical dislocation. The quadriceps (where artery ligation was performed) and the gastrocnemius (from the limb where artery ligation was performed) muscles were collected, frozen in liquid nitrogen and stored at − 80 °C until needed. Subsequently, multiple frozen sections were cut (5 μm thickness) on a cryostat and placed on glass slides.

### Histochemical staining

Tissue sections were prepared and stained with hematoxylin and eosin solution (Merck, Darmstadt, Germany) or with 0.1% solution of Fast Green and 0.1% solution of Sirius Red (Merck, Darmstadt, Germany) in 1.2% picric acid. The analysis of the specimens was conducted using the Nikon Eclipse 80i microscope (Nikon Instruments Inc., Melville, NY, USA). Histological measurements were taken in 5 randomly selected fields of each tissue section. The number of muscle fibers was calculated per mm^2^ of muscle. To analyze muscle regeneration, normal and regenerative muscle fibers (characterized by centrally located nuclei) were counted. Fibrosis area was calculated as the percentage of the Picrosirius red-stained collagen in the muscles per total area of tissue (sirius red- and fast green-stained tissue) using ImageJ 1.48v (NIH, Bethesda, MD, USA).

### Immunofluorescence staining

Sequentially, tissue sections were fixed in cold acetone or with 4% paraformaldehyde solution for 10 min, permeabilized with 0.1% (v/v) Triton X-100 (Merck, Darmstadt, Germany) in PBS for 10 min, then blocked with 2.5% normal goat serum (Vector Laboratories, Burlingame, CA, USA) for 30 min at room temperature and incubated with primary antibody at 4 °C overnight. After washing twice with PBST, slides were incubated with secondary antibody for 45 min at room temperature. Blood vessels were stained with anti-CD31 antibody (Abcam, Cambridge, UK) or fluorescein-labeled Griffonia simplicifolia lectin I (GSL I) isolectin B4 (Vector Laboratories, Burlingame, CA, USA). Pericytes were stained with anti-CD146 antibody (BioLegend, San Diego, CA, USA). Arteries were stained with anti-α-smooth muscle actin antibody (Abcam, Cambridge, UK). Identification of macrophages was performed using an anti-F4/80 antibody (Abcam, Cambridge, UK). Macrophages with M2 phenotype were stained with anti-CD206 antibody (BioLegend, San Diego, CA, USA). The appropriate secondary antibodies conjugated with Texas Red, FITC, or Alexa Fluor594 (Abcam, Cambridge, UK) were used. Nuclei were counterstained with DAPI (Vector Laboratories, Burlingame, CA, USA). Fluorescence imaging of the stained sections was performed with the confocal microscope LSM710 (Carl Zeiss Microscopy GmbH, Jena, Germany). Capillaries were counted in 10 randomly selected fields of each tissue section. The capillary density was calculated as the number of capillaries/mm^2^ of muscle. Arterioles were counted in 5 randomly selected fields of each tissue section. The average arteriole diameter was calculated by ImageJ 1.48v (NIH, Bethesda, MD, USA). The results expressed as the percentage of area (%) were calculated by ImageJ 1.48v (NIH, Bethesda, MD, USA).

### Statistical analysis

The normality of the distribution was verified with the Shapiro–Wilk test. Next, the equality of variances for variables was tested by Levene or Brown–Forsythe tests. For variables with normal distribution, the one-way ANOVA (with post hoc Tukey HSD test) was carried out (marked with the hashtag in the figures). Average values from 5 to 10 fields of view per animal (*n* = 3) were expressed as mean ± standard error (SE.) Otherwise, nonparametric test was performed (the Kruskal–Wallis followed by the post hoc multiple comparisons of rank sums test) (marked with an asterisk in the figures). For those, data values were presented as median, quartiles and min/max values as whiskers. The statistical analysis was performed using the Statistica 12 software.

## Results

### The influence of the method of ADSCs delivery on muscle regeneration in hindlimb ischemia

**Fig. 1:**
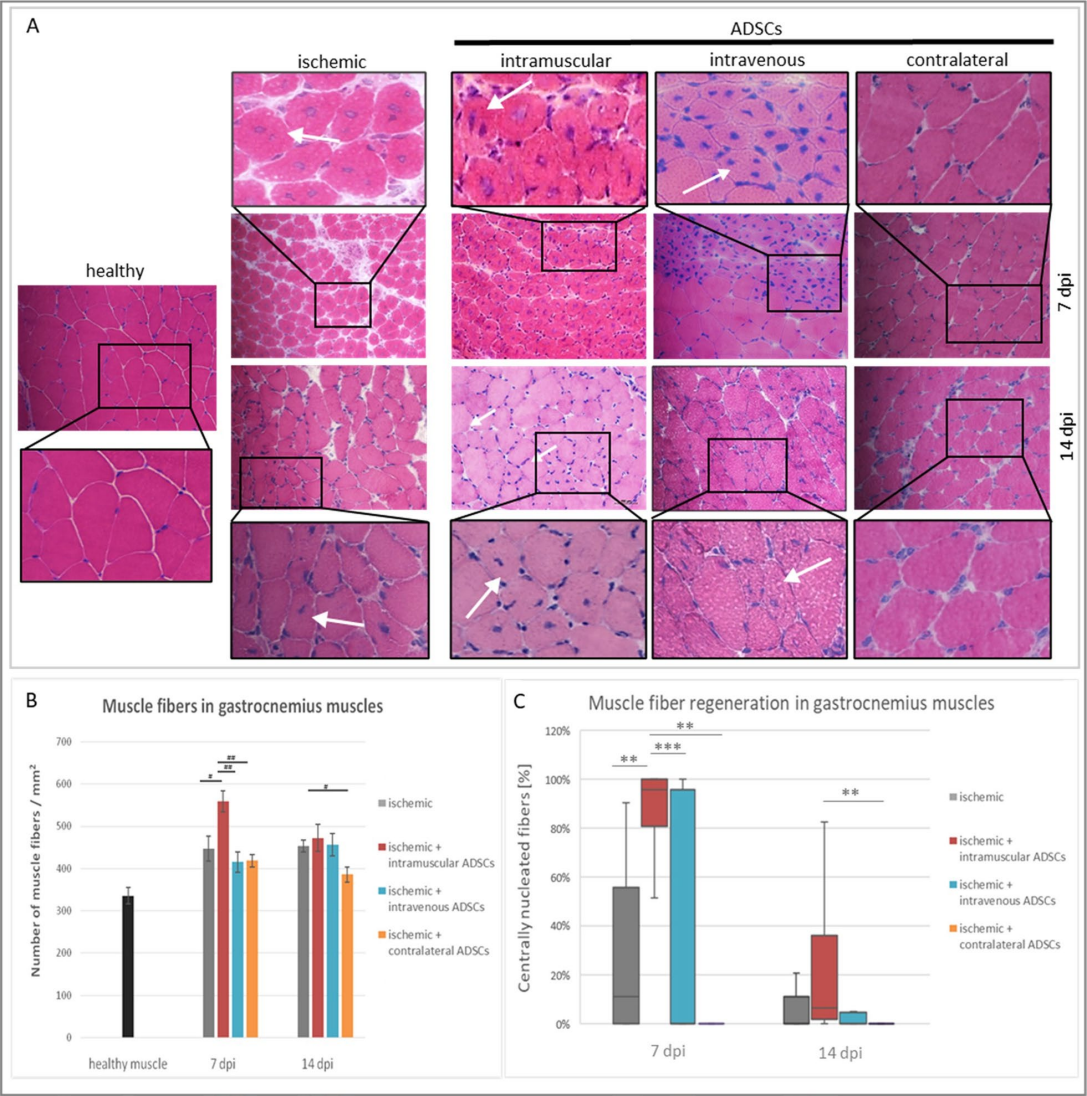
Muscle regeneration in hindlimb ischemia after ADSCs delivery. **A** Representative images of transverse sections of the gastrocnemius muscles stained by H&E in: healthy, ischemic, intramuscular (i.m.), intravenous (i.v.) and contralateral (c.) groups on the 7th and the 14th days post-injury (dpi). **B** Number of muscle fibers counted in collected muscles on the 7th and the 14th dpi. The number of muscle fibers was determined in 5 fields of view per animal (*n* = 3–5) at a magnification of 20× and calculated per 1 mm^2^ of tissue. **C** Ratio of regenerating myofibers (white arrows) counted in collected muscles on the 7th and the 14th dpi. Regenerating myofibers with two or more centralized nuclei was counted in 5 fields of view per animal (*n* = 3–5) at a magnification of 20×. Results are presented as mean ± standard error (SE) or in box plot as median, quartiles and min/max values as whiskers. #*p* < 0.02, ##*p* < 0.002 by the ANOVA followed by the Tukey’s post hoc test. **p < 0.02, ***p < 0.0001 evaluated with Kruskal–Wallis one-way analysis of variance and multiple comparison of mean ranks for all groups

To investigate the skeletal muscle fiber regeneration, histological analysis of muscles was carried out using hematoxylin and eosin staining (Fig. 1). Muscle regeneration was indicated as overall count of muscle fibers and regenerative muscle fibers. In the gastrocnemius muscles on the 7th day post-injury (dpi), more muscle fibers and greater muscle regeneration (small, basophilic muscle fibers with one or more centrally located nuclei) were observed when ADSCs were injected intramuscularly into ischemic limb (Fig. 1B, C). Additionally on the 14th dpi, an increase in regenerating muscle fibers was observed in the intramuscular group compared to the contralateral group (Fig. 1C). In the intravenous group, more regenerative muscle fibers were observed compared to the contralateral group; however, these results were not statistically significant. In group where ADSCs were injected into contralateral limb, no muscle regeneration was observed on the 7th and the 14th dpi.

### The influence of the method of ADSCs delivery on angiogenesis in hindlimb ischemia

**Fig. 2:**
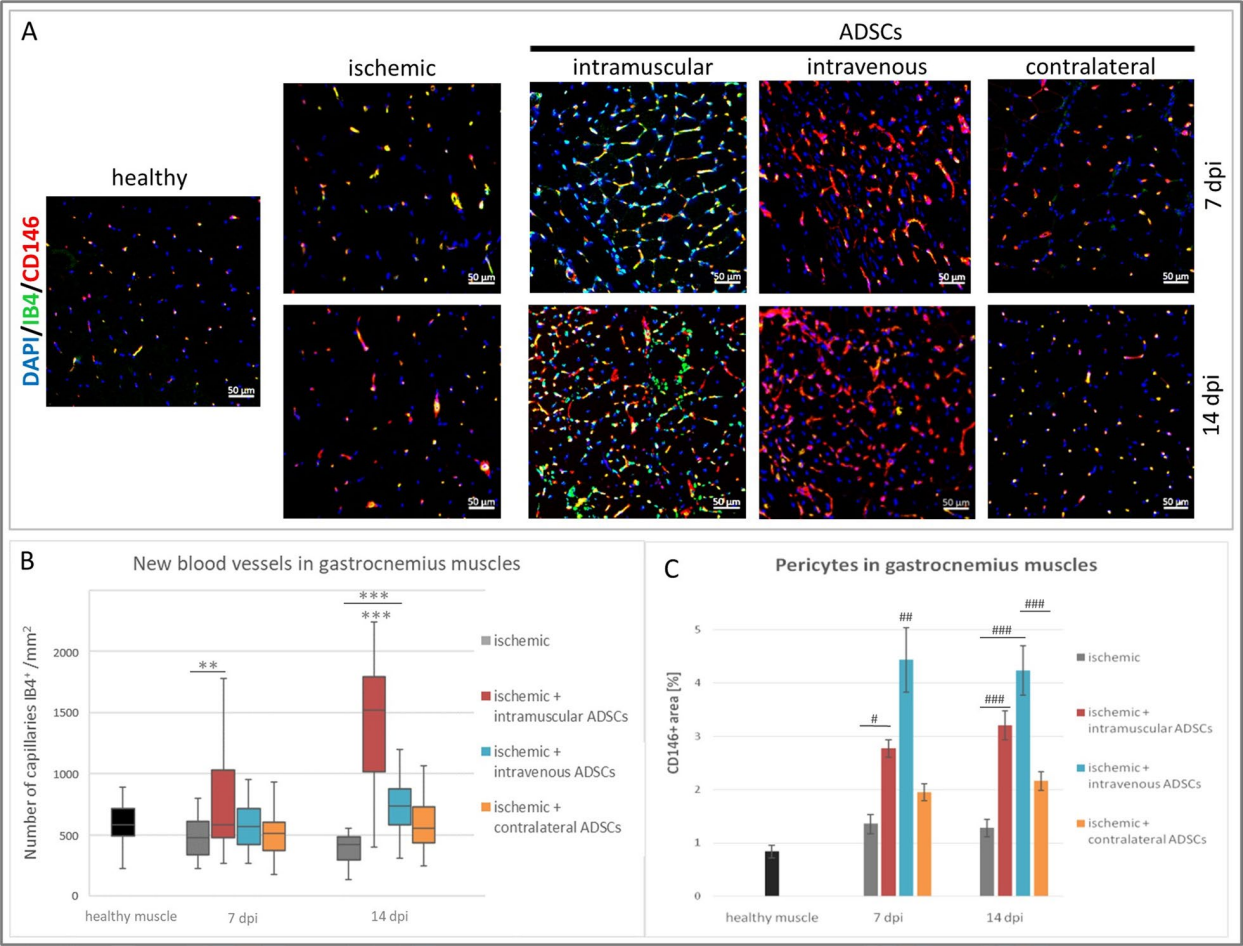
Angiogenesis in hindlimb ischemia after ADSCs delivery. **A** Representative images of mature blood vessels—double immunostaining of endothelial cells (IB4, green) and pericytes (CD146, red) in: healthy, ischemic, intramuscular, intravenous and contralateral groups on the 7th and the 14th days post-injury (dpi). Nuclei stained with DAPI (blue), scale bars = 50 μm. **B** Number of capillaries (IB4^+^) counted in collected muscles on the 7th and the 14th dpi. The number of capillaries (IB4^+^) in each group was determined in 10 fields of view per animal (*n* = 3–5) at a magnification of 40× and calculated per 1mm^2^ of tissue. **C** Percentage of the area occupied by pericytes (CD146^+^ cells) was counted in collected muscles on the 7th and the 14th dpi. The total area occupied by CD146^+^ cells was calculated using ImageJ software in 5 fields of view per animal (*n* = 3–5) at a magnification of 20× Results are presented as mean ± standard error (SE) or in box plot as median, quartiles and min/max values as whiskers. #*p* < 0.05, ##*p* < 0.005 ###*p* < 0.0001 by the ANOVA followed by the Tukey’s post hoc test. *p < 0.05, **p < 0.02, ***p < 0.001 evaluated with Kruskal–Wallis one-way analysis of variance and multiple comparison of mean ranks for all groups

In order to evaluate angiogenesis after ADSCs delivery, capillary density was assessed by immunofluorescence labeling with Griffonia simplicifolia lectin I (GSL I) isolectin B4. Immunofluorescent assessment of the gastrocnemius muscles on the 7th day post-injury showed more capillaries only after intramuscular ADSCs delivery (Fig. 2B). There was no difference in the number of blood vessels between ADSCs groups on the 7th dpi. Significant increase in the number of blood vessels was observed on the 14th dpi in the intramuscular and the intravenous groups compared to the control (ischemic) group. Additionally, twice more blood vessels were observed in the intramuscular group when compared with the intravenous and contralateral groups.

To assess the maturity of the formed blood vessels, endothelial cells and pericytes were labeled with Griffonia simplicifolia lectin I (GSL I) isolectin B4 and anti-CD146 antibody, respectively. In the gastrocnemius muscle, the significant increase in the number of mature vessels, where endothelial cells were covered with pericytes, was observed after intravenous ADSCs delivery in both 7th and 14th dpi. The significant increase in the number of mature vessels was also observed in the intramuscular group compared to control (ischemic) (Fig. 2C).

### The influence of the method of ADSCs delivery on arteriogenesis in hindlimb ischemia

**Fig. 3:**
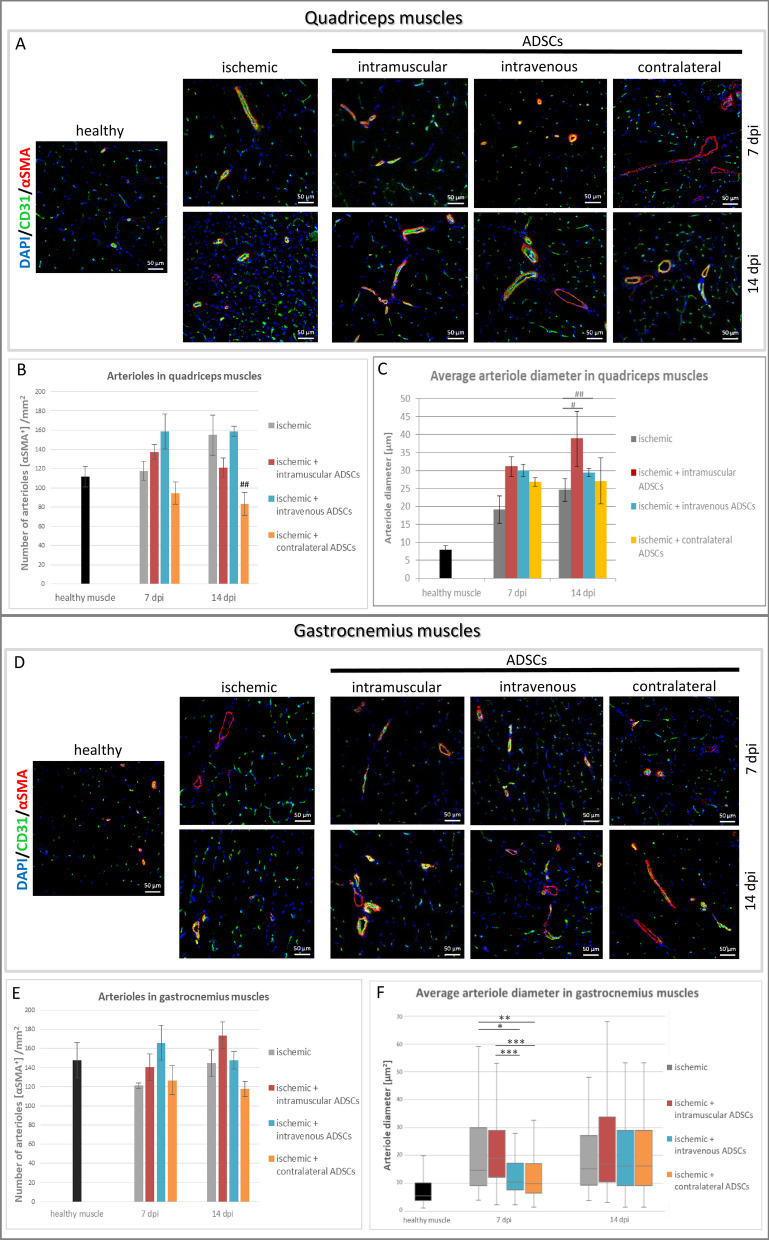
Arteriogenesis in hindlimb ischemia after ADSCs delivery. **A**, **D** Representative images of arterioles—double immunostaining of endothelial cells (CD31, green) and arterioles (αSMA, red) in: healthy, ischemic, intramuscular, intravenous and contralateral groups on the 7th and the 14th days post-injury (dpi). Nuclei stained with DAPI (blue), scale bars = 50 μm. **B**, **E** The number of arterioles (αSMA^+^ cells) was counted in collected quadriceps (**B**) and gastrocnemius (**E**) muscles on the 7th and the 14th dpi. The number of arterioles in each group was determined in 5 fields of view per animal (*n* = 3–5) at a magnification of 20× and calculated per 1mm^2^ of tissue. Each column in the graph represents a mean ± SE. **C**, **F** The average arteriole diameter was counted in quadriceps (**C**) and gastrocnemius (**F**) muscles collected on the 7th and the 14th dpi. The average arteriole diameter was calculated using ImageJ software in 5 fields of view per animal (*n* = 3–5) at a magnification of 20×. Results are presented as mean ± standard error (SE) or in box plot as median, quartiles and min/max values as whiskers. #*p* < 0.05, ##*p* < 0.005 by the ANOVA followed by the Tukey’s post hoc test. *p < 0.02, **p < 0.002, ***p < 0.0001 evaluated with Kruskal–Wallis one-way analysis of variance and multiple comparison of mean ranks for all groups

In order to evaluate arteriogenesis, we investigated the proximal (quadriceps) and the distal (gastrocnemius) muscles, due to the fact that arteriogenesis is mostly induced in proximal muscles. Arterioles were doubly stained using anti-CD31 and anti-α-smooth muscle actin (αSMA) antibodies. The analysis of quadriceps muscles showed an increase in the number of αSMA-positive arterioles formed after intramuscular and intravenous ADSCs delivery compared to the contralateral group on the 14th dpi (Fig. 3B). In the gastrocnemius muscles, no significant differences in the number of arterioles were observed in all groups (Fig. 3E). Furthermore, inside diameter of an artery was measured. The analysis of quadriceps muscles revealed that CD31- and αSMA-positive vessels after intramuscular and intravenous ADSCs delivery had a larger lumen diameter than arterioles observed in the control (ischemic) group on the 14th dpi (Fig. 3C). In the gastrocnemius muscles on the 7th dpi, arterioles’ diameter was significantly increased in the intramuscular and control groups compared to the intravenous and the contralateral groups. However, on the 14th dpi in the gastrocnemius muscle there were no significant differences in the lumen diameter of arterioles (Fig. 3F).

### The influence of the method of ADSCs delivery on the macrophage infiltration in hindlimb ischemia

**Fig. 4:**
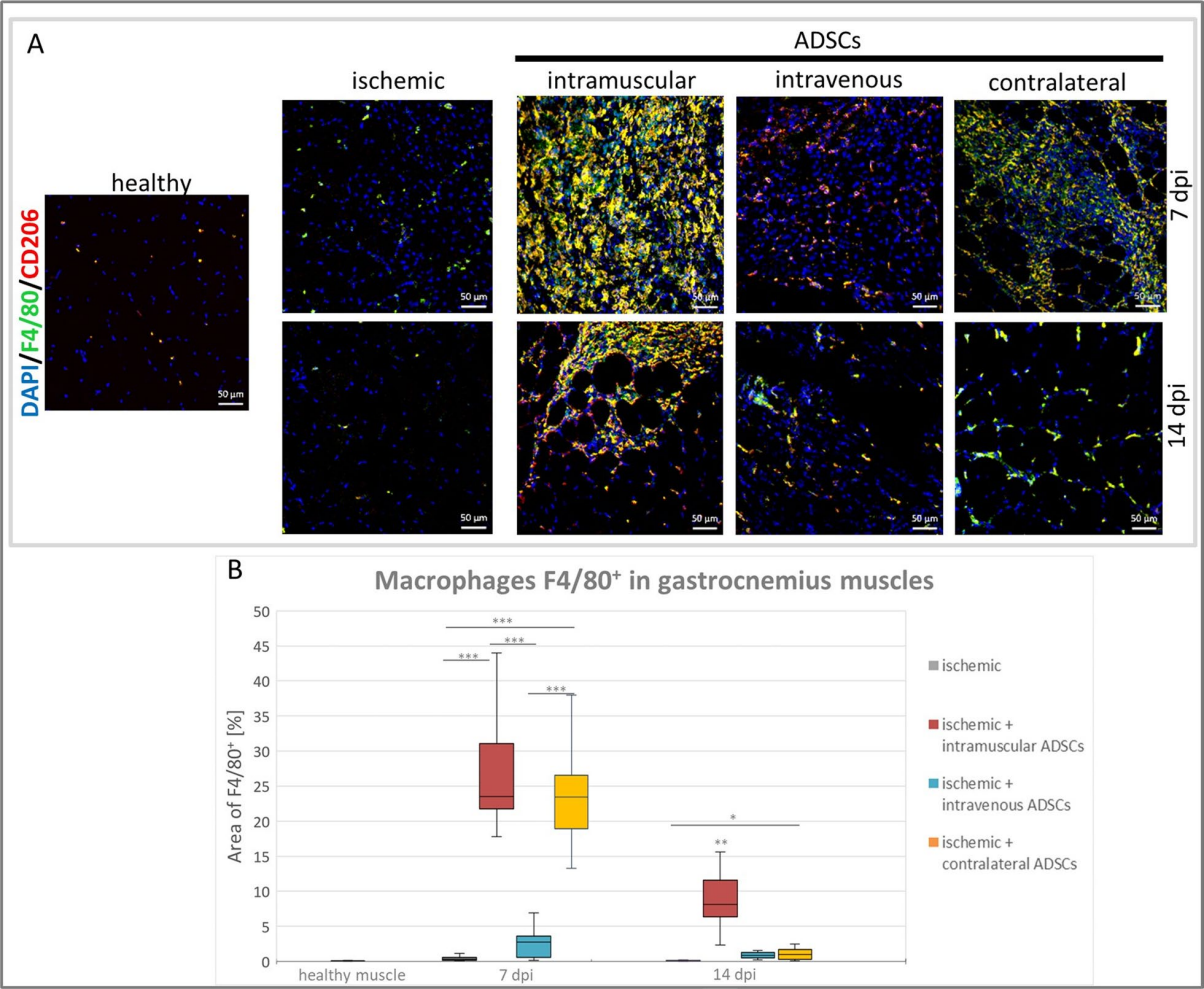
Influx of macrophages in hindlimb ischemic tissue after ADSCs delivery. **A** Representative images of M2 macrophages—double immunostaining of [F4/80^+^ (green), CD206^+^ (red)] in: healthy, ischemic, intramuscular, intravenous and contralateral groups on the 7th and the 14th days post-injury (dpi). Nuclei stained with DAPI (blue), scale bars = 50 μm. **B** Percentage of area occupied by macrophages (F4/80^+^ cells) was counted in collected muscles on the 7th and the 14th dpi. The total area occupied by F4/80^+^ cells was calculated using ImageJ software in 5 fields of view per animal (*n* = 3–5) at a magnification of 20×. Results are represented in box plot as median, quartiles and min/max values as whiskers. *p < 0.05, **p < 0.005, ***p < 0.0001 evaluated with Kruskal–Wallis one-way analysis of variance and multiple comparison of mean ranks for all groups

In order to evaluate the influx of anti-inflammatory, proangiogenic M2 macrophages, infiltrating cells in muscles were doubly stained using anti-F4/80 and anti-CD206 antibodies. The highest area occupied by macrophages (cells expressing F4/80) was observed in the group where ADSCs were delivered intramuscular on both the 7th and the 14th days post-injury. Additionally, an increase in the area of F4/80-positive cells was also observed in the contralateral group compared to the control group (Fig. 4B).

## Discussion

Currently, many researches are underway using mesenchymal stromal cells as a promising strategy in the therapy of critical limb ischemia [7–9, 17]. The greatest interest of these cells results from their properties: They reduce inflammation, affect tissue regeneration and initiate the formation of new blood vessels [18]. Mesenchymal stromal cells derived from various sources such as bone marrow, fetal membrane, umbilical cord blood and adipose tissue have been shown to be beneficial in animal models of critical limb ischemia [8, 9, 19]. Nevertheless, it has been stated that adipose-derived stromal cells (ADSCs) show greater proangiogenic effect than bone marrow-derived mesenchymal stromal cells (BM-MSCs) in ischemic disease therapy [10, 12, 13].

However, there are still many inaccuracies regarding the site of the injection, the timing and the appropriate number of cells delivered in therapy of critical limb ischemia. In several preclinical studies, ADSCs have been delivered by intravenous injection [20], while in others an intramuscular injection method has been used [10, 21–24]. Nevertheless, none of these studies have presented a comparative analysis of the best method of mesenchymal stromal cell transplantation for the critical limb ischemia treatment. Therefore, we focused on the therapeutic efficacy of different delivery methods of adipose-derived mesenchymal stromal cells in a murine model of hindlimb ischemia.

Our research revealed that muscle regeneration, angiogenesis, arteriogenesis and macrophage infiltration in murine model of hindlimb ischemia differ depending on the ADSCs delivery method. We demonstrated that intramuscular route (directly into ischemic limb) of ADSCs delivery is more efficient in functional recovery after critical limb ischemia than intravenous or contralateral route.

In this study, a protocol developed by Brenes et al. [25] was used, in which the hindlimb femoral artery was ligated with surgical sutures in two places, which causes distal ischemia in the gastrocnemius muscle [26]. Ligation of the femoral artery without removal of the mouse model more closely reflects critical limb ischemia in humans, in which the arteries are occluded but still present. The study was performed on the immunocompetent C57BL/6NCrl mouse strain, due to the fact that the immune system plays an essential role in the process of vascular remodeling [27].

In our previous work, we observed that human ADSCs induced better therapeutic effects than allogeneic ADSCs in the immunocompetent mouse strain. Moreover, our previous results showed that the therapeutic effect of human ADSCs is dependent on the type of injected mesenchymal cells, not by itself xenograft. There was also no therapeutic effect observed after delivery of control human fibroblasts [10]. Experiments were carried out on 8–12-week-old animals, due to the fact that the younger ones quickly regain the normal functioning of the limb, which makes impossible to study the effects of proangiogenic factors [26]. Due to the fact that only a small part of the administered cells survives and gives a therapeutic effect, it is important to determine the appropriate number of cells administered into the damaged muscle observed that increasing the number of administered cells from 5 × 10^5^ to 1 × 10^6^ cells improved the blood supply to the limb. This dependence was not observed after increasing the number to 2 × 10^6^ cells [25].

The method of cells delivery seems to play a crucial role in therapeutic efficacy of MSCs. There are two main methods of cells introducing into the body: local delivery and systemic delivery [18]. Topical application of MSCs is the least invasive method for cells delivery and shows great potential in the treatment of ischemic wounds. Most reported to date preclinical studies have transplanted MSCs via the intramuscular route [28–30]. Skeletal muscles as a place of MSCs delivery have many advantages, such as extended dwell time provided by dense muscle fibers trapping MSCs in situ [28]. Moreover, high vascular density of the muscles provides an opportunity for the systemic release of MSCs factors. Additionally, high abundance of tissue provides multiple injection sites [31]. Beegle et al. [32] using optical imaging showed that BM-MSCs delivered into hindlimb ischemia were detected in the muscle up to 21 days post-transplantation, which was also confirmed by our previous research [10].

In the present work, intramuscular injection was one method of ADSCs administration. Cells were delivered into the gastrocnemius muscle of limb where femoral artery ligation was performed. Due to the fact that ligation of the femoral artery causes distal ischemia in the gastrocnemius muscle [26], ADSCs were delivered into this muscle. Our next method was intravenous ADSCs injection through the tail vein, which is an equally popular method of cells delivery [18]. The most studies that examine homing and engraftment of mesenchymal stromal cells via systemic administration have shown that the majority of administered cells (~ 80%) accumulate directly in the lungs and are cleared within few days [33]. This is due to the fact that cultured MSCs with estimated diameter of 30 μm are entrapped in the micro-capillaries of lungs [34]. In addition, it is noticed that factors secreted by ADSCs are released into the bloodstream and therefore may have a systemic effect [18]. Therefore, our next method of ADSCs delivery was intramuscular injection into the gastrocnemius muscles of contralateral, healthy limb. This method could be effective in the treatment of patients, e.g. with a myocardial infarction, for which it is difficult to apply the direct administration method. This would avoid the administration of cells to damaged tissues such as the heart muscle or the ischemic limb. Additionally, administration to healthy muscle appears to be a better administration site when multiple cell administrations are required. ADSCs are a kind of “reactor” that constantly produces and secretes factors into the blood stream [14]. Therefore, a healthy muscle could be a better place for cells to hold and release factors than a damaged muscle or intravenous route. Therefore, in our work, we investigate whether administration to a healthy limb will bring a better therapeutic effect than administration to a damaged muscle.

Muscle tissue is most vulnerable to damage in the ischemic limb [35]. Muscle-specific unipotent stem cells, i.e. satellite cells, play a major role in a skeletal muscle repair. In response to signals from the environment of the damaged muscle, satellite cells are activated. Activated cells enter into cell cycle and differentiate into proliferating myoblasts and ultimately myocytes. Myocytes are assembled to create myotubes that mature into new functional muscle fibers to replace damaged ones [36]. Regenerative muscle fibers contain one or more centrally located nuclei [37]. Here we presented that ADSCs delivered directly to the ischemic muscle significantly increase the regeneration of muscle myofibers already on 7th dpi. Our group [10] and others [37–39] stated before that ADSCs promote muscle regeneration; however, their mechanism is still unclear. ADSCs have also potentiality for myogenic transdifferentiation in vitro [40] and in vivo [41, 42]. In turn, other researchers did not observe direct participation of the implanted ADSCs in new myofiber formation [38]. Therefore, the major effect of MSCs seems to be related to MSCs ability to secrete cytokines and growth factors.

For the proper maturation of myotubes and regeneration of the damaged muscle, vascular reconstruction is required [43]. Therefore, in our work we investigated the formation of new blood vessels after ADSCs injection depending on the site of cell administration. Analysis of gastrocnemius muscles showed a significant increase in blood vessel formation on the 7th dpi in the intramuscular ADSCs group. After intravenous ADSCs delivery, we also observed more blood vessels compared to the control (ischemic) group, but not as much as in the intramuscular group.

Pericytes are located on the endothelial cells in normal, mature capillaries and are necessary for proper blood flow in the formed blood vessels [44]. In our work, we observed greater area occupied by pericytes in the group that received the cells intravenously compared to the intramuscular route. However, it should be noted that some pericyte subpopulations may be a source of collagen-producing cells which contribute fibrosis in skeletal muscle [45]. Further, type 1 pericytes contribute to fat accumulation which increases dysfunction of muscle tissue [46]. Therefore, the exact role of pericytes in ischemic muscles requires further understanding and investigation.

It should be noted that angiogenesis itself has a limited capacity to enhance the perfusion of the surrounding ischemic tissue [47]. It has been reported that only collateral arteries are essentially capable of providing a sufficiently large blood flow to ischemic areas at risk of necrosis or loss of function [48]. Therefore, in our work we investigated the route of ADSCs administration on arteriogenesis. Additionally such analysis was carried out in proximal to the ischemic regions—quadriceps muscles (Scheme 1D). Arteriogenesis is characterized by the growth, development and remodeling of preexisting vessels, mainly arterioles, into larger collateral arteries [47]. Our results indicate that intramuscular ADSCs delivery increased the size of the arteries formed already on day 7th compared to the others ADSCs delivery groups.

Inflammation and recruitment of immune cells, mainly macrophages and lymphocytes, are an integral part of muscle regeneration and neovascularization in hindlimb ischemia therapy [14]. In our previous works, we have demonstrated that after intramuscular ADSCs delivery, macrophages that infiltrate into ischemic limb have mainly M2—proangiogenic phenotype [10, 14]. Numerous studies have shown that MSCs interact with macrophages and regulate their functions mainly by differentiating them toward M2 phenotype [49–52]. Furthermore, ADSCs promote changes in the morphology of infiltrating macrophages and their tight relationship with blood vessels formation [14]. The presented results indicate that the highest influx of macrophages (F4/80^+^) was observed in the intramuscular group. Almost all F4/80^+^ macrophages were CD206 positive, indicating their M2 phenotype (Additional file 1: Fig. S1). We also observed a large influx of macrophages in contralateral group on the 7th dpi. However, on the day 14th the influx was not observed as in the intramuscular group. These results may indicate that local administration of ADSC into diseased muscle prolongs the therapeutic effect. After intravenous ADSCs delivery, no influx of macrophages in muscles was observed, which is probably due to the fact that most of the intravenous injected MSCs are phagocyted by lungs macrophages, which in turn acquire a M2 phenotype [33].

In conclusion, in the present study we have noticed that injection of ADSCs directly into ischemic limb increases: (1) muscle fiber regeneration, (2) the number of capillaries and (3) the influx of F4/80^+^/CD206^+^ macrophages. It seems that it is the optimal delivery method of ADSCs in hindlimb ischemia therapy. In our opinion, a combined treatment using intramuscular (into ischemic limb) and intravenous administration of ADSCs could bring more medical benefit. The combination of these two routes of administration could further improve the vascularization of the ischemic muscle. Moreover, such a combination in humans would offer the possibility of increasing the amount of administered cells.

This study has some limitations. We did not compare the mechanisms of action of these three methods of ADSCs delivery. The more detailed mechanism of ADSCs involvement in ischemic limb regeneration should be investigated.

## Conclusions

For the first time, our study showed a direct comparison of adipose-derived stromal cells (ADSCs) delivery methods for the treatment of murine hindlimb ischemia. In our work, we have noticed that injection of ADSCs directly into ischemic limb increased: (1) muscle fiber regeneration, (2) the number of capillaries and (3) the influx of F4/80^+^/CD206^+^ macrophages in proximal, gastrocnemius muscles. We demonstrate that ADSCs delivery via the intramuscular route (directly into ischemic limb) is more efficient for functional recovery after critical limb ischemia than intravenous ADSCs delivery and intramuscular into contralateral limb routes.

### Supplementary Information

**Additional file 1.** Materials and Methods: Animals. **Figure S1.** Separate fluorescence channels of images of M2 macrophages.

## Data Availability

Not applicable.

## Abbreviations

ADSCs: Adipose-derived mesenchymal stromal cells
CLI: Critical limb ischemia
MSCs: Mesenchymal stromal cells
BM-MSCs: Bone marrow-derived mesenchymal stromal cells
