# Supplementary material for: The comparison of adipose-derived stromal cells (ADSCs) delivery method in a murine model of hindlimb ischemia
Source: Stem Cell Res Ther. 2024 Feb 2;15:27. doi: 10.1186/s13287-024-03634-2 (PMC10836003; doi:10.1186/s13287-024-03634-2)
Supplement: Supplementary file 1 — Additional file 1. Materials and Methods: Animals. Figure S1. Separate fluorescence channels of images of M2 macrophages. [file 13287_2024_3634_MOESM1_ESM.doc]

**Materials and Methods: Animals**

All mice were housed in the Maria Sklodowska-Curie National Research Institute of Oncology, Gliwice Branch (Poland). The animals were bred in accordance with standards in sterile, individually ventilated cages, in shelves equipped with air supply and exhaust units (integrated at the top of the shelf with HEPA-filtered Allentown’s IVC System (Allentown Caging Equipment Co, NJ, USA) and temperature and humidity sensors. The temperature in the animal house were maintained at a constant level of 22°C ± 2°C, humidity at 50-60%. Light cycle 12 hours (12/12), air exchange were maintained at 12-20 times per hour. The mice had constant access to sterile water and feed (Total Pathogen Free maintenance feed from Altromin (Altromin 1314, Altromin Spezialfutter GmbH & Co. KG, Germany)). Mice were kept in a cage with aspen wood bedding additionally enriched with lignin intended for nest construction. Qualified technical staff provided appropriate care depending on your needs. The general health of the animals were monitored by a veterinarian.

This study was carried out in strict accordance with the recommendations in the Guide for the Care and Use of Laboratory Animals of the National Institutes of Health. All experiments on animals were conducted in accordance with the 3R rule. Mice in experiment (eight-to-twelve-week old males with weight= 22g) were kept individually in a cage. Experiments were not blinded. All mice intended to the experiments were used. There were no exclusions.

Before starting the procedure, the micewere anesthetized with 2% isoflurane (MiniVent Model 845, Harvard Apparatus, USA).The limb were shaved and the surgical site were washed with iodine. Then the skin from the inner side of the left hindlimb was incised and the superficial femoral artery was exposed. The artery was ligated at two points using triple surgical knots. Skin incisions were closed and again were washed with iodine. Immediately after the procedure, an analgesic (Bunondol 0,1 mg/kg) were administered into the peritoneum. The analgesic were administered twice a day, every 12 hours.


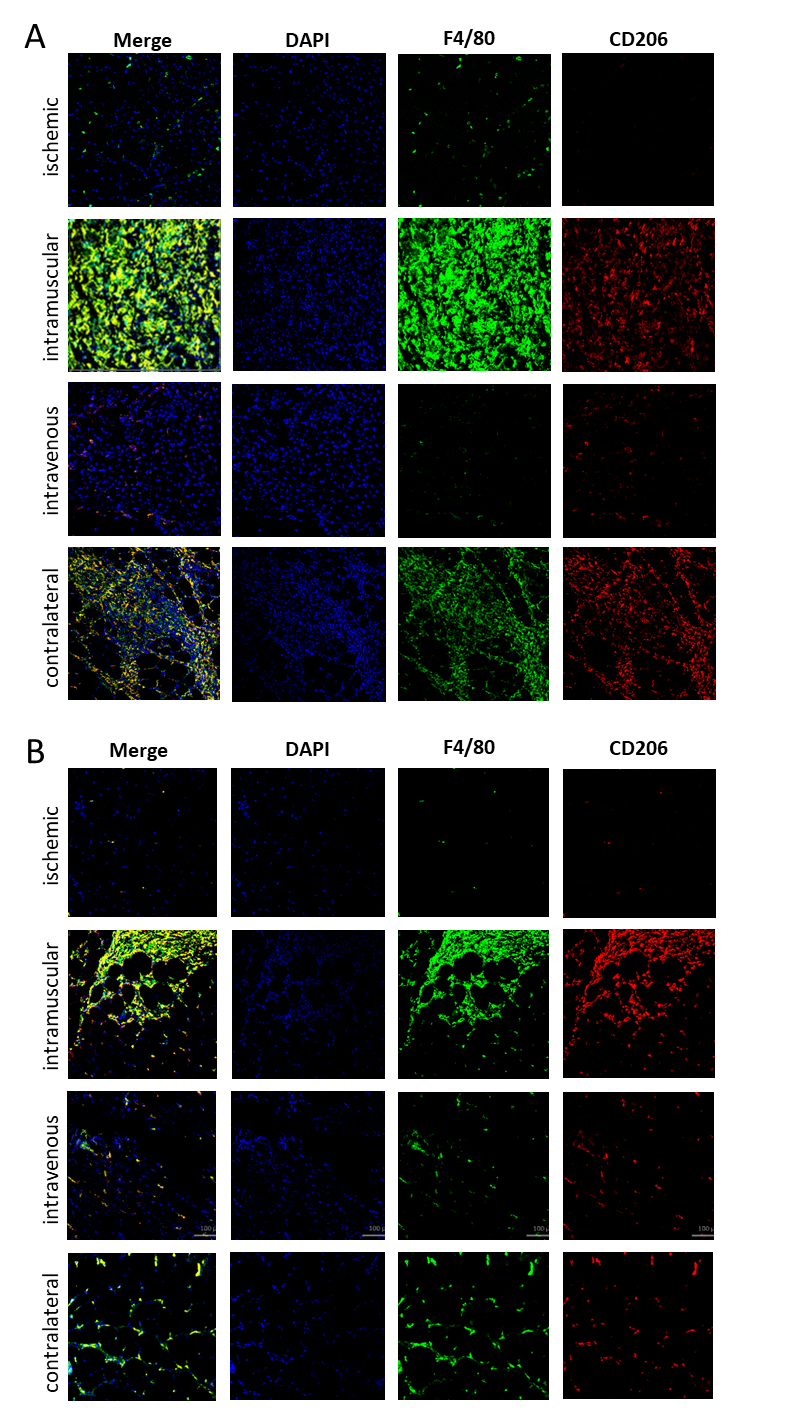


S.1 The influx of macrophages in hindlimb ischemic tissue after ADSCs delivery. The separate fluorescence channels of images of M2 macrophages - double immunostaining of (F4/80^+^ (green), CD206^+^ (red)) in: ischemic, intramuscular, intravenous and contralateral groups on the 7^th^ (A) and the 14^th^ (B) days post injury (dpi). Nuclei stained with DAPI (blue), scale bars = 50 μm.
